# UKRAINIAN WAR TRAUMA PATIENTS ABROAD: THE REHABILITATION PROCESS IN LIGHT OF LANGUAGE BARRIERS, CULTURAL DIFFERENCES, WAR, AND INFECTION ISOLATION

**DOI:** 10.2340/jrm.v57.42929

**Published:** 2025-08-11

**Authors:** Maria Ryssdal KRABY, Mariia TOROPCHYNA, Anders HOLTAN, Frank BECKER

**Affiliations:** 1 Institute of Clinical Medicine, University of Oslo, Oslo, Norway; 2 Sunnaas Rehabilitation Hospital, Nesodden, Norway; 3 Bila Tserkva City Hospital 2, Bila Tserkva, Ukraine; 4 National Coordination Centre for Medical Evacuation, Oslo University Hospital, Oslo, Norway

**Keywords:** armed conflicts, communication barriers, culturally competent care, multiple trauma, patient isolation, patient participation, rehabilitation

## Abstract

**Objective:**

To study the rehabilitation of Ukrainian war trauma patients abroad, focusing on 5 areas of particular interest: communication, cultural differences, infection isolation, psychosocial load, and the rehabilitation process.

**Design:**

Observational study.

**Subjects:**

(*i*) 14 Ukrainian patients who underwent rehabilitation at Sunnaas Rehabilitation Hospital, Norway; (*ii*) 15 members of multidisciplinary teams providing war trauma rehabilitation.

**Methods:**

Combined methods. Patients: medical file review, Client Satisfaction Questionnaire 8, custom questionnaire on the 5 focal areas. Hospital staff: focus-group interviews.

**Results:**

Patients preferred professional interpreters, while multilingual staff served an additional role in providing psychological support and cultural mediation. All patients experienced infection isolation, and health professionals worried about the added psychological strain this entailed. Patients reported high trust in the therapists and high satisfaction with rehabilitation. Although war and infection isolation were negative influences, patients reported overall good mood. Health professionals reported becoming more skilled at facilitating rehabilitation under these conditions.

**Conclusion:**

Despite challenges within the 5 areas assessed, providing rehabilitation to patients evacuated from a country at war is feasible and valuable for patients and health professionals. Both patients and health professionals showed willingness to adapt to foreign concepts, perhaps aided by multilingual health professionals acting as cultural mediators.

## LAY ABSTRACT

Sunnaas Rehabilitation Hospital provides rehabilitation to patients evacuated from Ukraine due to the ongoing war. Rehabilitation may be complicated when patients and hospital staff speak different languages and have different cultural backgrounds and views on how rehabilitation should be performed. Notably, the war may worsen patients’ psychological and social factors. Patients undergo infection isolation if carrying multidrug-resistant organisms. This study assessed how these potential sources of friction influenced rehabilitation from the perspectives of patients and health professionals. Both groups displayed flexibility and willingness to adapt to the challenges described herein, and health professionals who spoke both languages bridged gaps between them. Providing rehabilitation to patients evacuated from a country at war was feasible and valuable for patients and health professionals, and patients reported high levels of satisfaction. These experiences can benefit a vast group of patients, and they are important for civil protection.

After the Russian attack on Ukraine in February 2022, the European Union (EU) rapidly established a system for the medical evacuation of Ukrainian patients requiring specialized healthcare. Known as the MEDEVAC programme, it is part of the EU Civil Protection Mechanism (1). Of the 4,005 patients evacuated by 10 January 2025, 2,808 were trauma patients. Sunnaas Rehabilitation Hospital (SRH) near Oslo, Norway, provided specialized rehabilitation to 24 MEDEVAC patients who suffered traumatic head injury (TBI), spinal cord injury (SCI), and polytrauma with or without amputation.

Rehabilitation provided abroad may be influenced by potential sources of friction. First, rehabilitation and patient outcomes may suffer when patients and health professionals speak different languages (2–4). Although patients have the right to a professional medical interpreter (PMI) (5), few studies have investigated the extent of PMI use during inpatient rehabilitation (6).

Second, cultural differences might exist (e.g., between Ukraine and Western Europe). Ukrainians traditionally reported a low degree of trust in social institutions (7), although this has improved over the last years (8). Although currently undergoing a health reform, Ukraine’s healthcare system has long been based on a Soviet model characterized by inefficiency and corruption, lack of modernization, and not considering the population’s needs (9).

Third, infection isolation during hospitalization may negatively influence rehabilitation (10, 11). Ukraine has a high prevalence of multidrug-resistant organisms (MDROs) (12), which worsened due to war (13). The European Centre for Disease Prevention and Control recommends multimodal infection prevention and control strategies for patients who have been hospitalized in Ukraine, including the isolation of MDRO carriers (12).

Fourth, patients from Ukraine may experience persistently increased psychosocial load due to the ongoing war (14, 15). This may be exacerbated by stigma related to mental illness in Ukraine, which faces insufficient mental health awareness and prevention, as well as high rates of alcohol abuse and suicide (16).

Fifth, rehabilitation in Ukraine has long lacked systematic coordination and a national strategy, with gaps in the continuum of care. Moreover, several rehabilitation professions are not licensed or do not exist (17). The focus has typically been on providing patients with a pension rather than improving their function and participation (18). In recent years, several initiatives have been launched to improve rehabilitation (19). However, Ukrainians still associate rehabilitation with post-Soviet practices (e.g., health resorts, sanatoriums, massage therapy) (17). This may lead to different expectations than active rehabilitation based on the International Classification of Functioning (ICF) (20, 21).

Globalization makes these 5 potential sources of friction increasingly relevant in rehabilitation. This combined methods study aimed to describe the Ukrainian MEDEVAC patient group and the rehabilitation that they received at SRH from the perspectives of patients and health professionals. We aimed to shed light on 5 specific areas of importance when providing rehabilitation abroad: communication, cultural differences, infection isolation, psychosocial load, and the rehabilitation process.

## METHODS

### Study participants

This observational study had 2 participant groups. The first comprised adult Ukrainian citizens evacuated to Norway through MEDEVAC who underwent rehabilitation at SRH. Exclusion criteria were age < 18 years, and inability to remember rehabilitation due to cognitive impairment. Following advice from the regional ethics committee, 1 potential patient participant was not approached due to personal circumstances. Ukrainian MEDEVAC patients were identified through a unique code in the patient administrative system. A nursing assistant who spoke Norwegian and Ukrainian made initial contact. Hospitalized patients were invited to participate in person. Based on the available contact information, discharged patients were contacted by telephone, e-mail, or mail.

The second group comprised multidisciplinary team (MDT) members with experience in the rehabilitation of Ukrainian MEDEVAC patients at SRH. We aimed to ensure the representation of all relevant professions and departments. Hospital staff in relevant departments received an e-mail invitation. Based on their replies, purposive quota sampling was performed to ensure the participation of key personnel with particular expertise and a representative group composition (22).

All participants received an information and consent form to sign prior to participation, which was translated into Ukrainian by a professional translator and given final approval by a Ukrainian physical medicine and rehabilitation (PMR) physician.

### Data collection

Data were collected in 3 ways: medical file review, patient questionnaires, and focus-group interviews.

*Medical files*. Medical files were reviewed to retrieve data on patient background and injury, nutrition, medical complications, MDRO carriership, isolation regimen, the rehabilitation process and post-discharge needs. One researcher (MRK) read all medical and multidisciplinary reports. Searches for the following medical complications during rehabilitation were performed: emergency admissions to another hospital, patient falls, infections requiring treatment, thrombosis incidents, and bladder and bowel issues.

*Patient questionnaires.* Patient perspectives were collected through 2 questionnaires, which were translated into Ukrainian by a professional translator and given final approval by a Ukrainian PMR physician.

The Client Satisfaction Questionnaire 8 (CSQ-8) comprises 8 questions to assess how satisfied patients were with the received treatment (23–25). Based on 4 response categories, each question provides a score from 1 to 4. The total score ranges from 8 to 32, with a higher score indicating a higher level of treatment satisfaction.

The second questionnaire was custom-made to assess our 5 domains of interest (see Appendix S1) and developed with a Ukrainian PMR physician to consider cultural differences. Thirty-eight questions were multiple choice and 10 asked for a comment. Some questions were extracted from the patient satisfaction questionnaire given to all SRH patients. Most patients (12/14) were treated in the same department. Therefore, participants’ answers could be compared with the replies of other patients in that particular department in 2023. Questionnaires were presented by the multilingual nursing assistant or the first author using telephone PMI, so that participants could ask questions if necessary.

*Focus-group interviews.* To assess the perspectives of health professionals, semi-structured focus-group interviews were conducted, with a focus on the 5 main themes. Additionally, health professionals were asked whether they thought the rehabilitation provided was useful for patients, what worked particularly well, what they would have done differently, and how the rehabilitation developed over time. This form of qualitative data collection was chosen to explore the experiences and perspectives of health professionals in their own words while allowing for clarification and further questioning when necessary (26, 27). One facilitator (MRK) was present during the interviews, which lasted for approximately 1 hour. Interviews were video recorded upon participant consent and transcribed verbatim.

### Data analysis

All data were handled confidentially and stored in an encrypted folder. Descriptive information from medical files is presented in tables. Absolute values were preferred due to the low sample size. Time and weight indications are presented as medians with ranges. Descriptive information from the questionnaires is presented in stacked bar charts with absolute values. Free-text comments are supplemented in the text.

Data from focus-group interviews were analysed inductively. Following transcription, statements were sorted according to the 5 main categories. Thereafter, sub-categories were identified within the main categories (28). The main findings are reported in the text.

## RESULTS

### Recruitment of participants

**Fig. 1:**
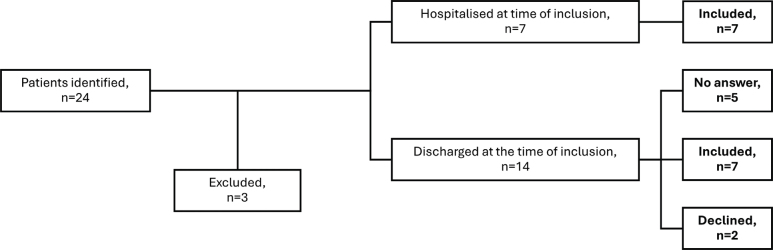
Patient recruitment. Reasons for exclusion were age < 18 years (n = 1), and inability to remember rehabilitation due to cognitive impairment (n = 1). One potential participant not approached due to personal circumstances.

By 12 November 2024, 24 Ukrainian MEDEVAC patients treated at SRH were identified (Fig. 1). Three of these were excluded in advance and 2 did not wish to participate. All 5 patients that we were unable to reach had partly missing contact information in their hospital files. Fourteen consented to participate, and 7 were hospitalized at the time. Overall, 15 health professionals were recruited.

### Participant characteristics

All patient participants except 1 were men (Table I). Twelve were soldiers, but only 3 were professional military personnel before the war. Except for 2, all had manual professions. Median age was 38 years (range: 26–48). All had war trauma, which mostly comprised multiple orthopaedic injuries. Twelve were treated in the same department. Median length of stay was 106 days (range: 59–286). Of the 13 who replied to the questionnaires, 2 did so in the same week they were discharged, while 4 had more than a month of rehabilitation remaining. The median number of days at SRH at the time of the survey was 92 (range: 31–265). Seven were directly discharged from SRH to an asylum reception centre (ARC), 5 were discharged to a permanent residence, and 1 was discharged to Ukraine. One was still hospitalized during data collection.

**Table I:** Descriptive characteristics of the 14 participating patients

Age in years, median (range)	38 (26–48)
Male	13/14
Soldier	12/14
Has children	9/14
War trauma	14/14
Blast injury	7/14
Mine injury	4/14
Missile/drone/rocket attack	3/14
Main diagnosis	
Amputation	7/14
Other orthopaedic trauma	5/14
Traumatic brain injury	1/14
Spinal cord injury	1/14
Length of stay at SRH in days, median (range)^a^	106 (59–286)
Number of months from injury until inclusion in the study, median (range)	16 (4–23)
Included in the study prior to final discharge	8/14
Number of days spent at SRH at the time of inclusion, median (range)	92 (31–265)
Number of months from final discharge until inclusion in the study, among those who had been discharged, median (range)	9 (2–16)
Discharge destination from SRH	
Not discharged from hospital	1/14
Asylum reception centre	7/14
Permanent residence in Norway	5/14
Ukraine	1/14

a One person was not discharged from hospital at the time of data analysis.

Health professionals were divided into 2 focus groups. Each focus group comprised a PMR physician, nurse, physical therapist, occupational therapist, and team coordinator. One also had a social worker and a dietitian. The other included a nursing assistant, psychologist, and department head. Each group contained 2 men.

### Rehabilitation provided

SRH offers specialized patient-centred rehabilitation programmes, accredited according to the international standards of the Commission on Accreditation of Rehabilitation Facilities. All patients had a private room and bathroom. Nursing staff were always available in the ward and a physician at the hospital. The MDT of every patient comprised a nurse, physician, physical therapist, occupational therapist, psychologist, social worker, and team coordinator. Other professions were involved if necessary (e.g., dietitians and orthopaedic engineers). Therapy sessions occurred during the workday. Weekends and evenings involved group activities and individual exercise opportunities. The rehabilitation process was individualized, goal-driven, and included meetings with the MDT. There, patients were actively involved in discussing long- and short-term goals, evaluating progress and planning life after discharge. The hospital almost exclusively used video or telephone PMIs.

MDRO carriers were in contact or droplet isolation while hospitalized (29). Although they generally had to remain in their rooms when in hospital, many could go outdoors directly from their rooms. During autumn 2022, infection isolation regimens were altered so that carriers of some MDROs could be in common areas and gyms, provided they were accompanied by hospital staff to ensure adherence to infection prevention guidelines.

### Medical complications

Table II displays a list of medical complications during rehabilitation. Four patients had emergency admissions to another hospital due to orthopaedic infection. One patient had a second admission due to a pertrochanteric fracture after falling on the amputation stump. Two of the 4 patients who fell during rehabilitation required surgery. Six patients developed infections: orthopaedic (*n* = 4), urinary tract (*n* = 1), oral fungal (*n* = 1), COVID-19 (*n* = 1), and external otitis (*n* = 1). Four were underweight upon arrival, and 8 were attended to by a dietitian. While the 2 patients with TBI and SCI lost weight during hospitalization, the others gained weight.

**Table II:** Medical information concerning the 14 patients during hospitalization

Number of emergency admissions to hospital, *n*	5
Infection	4
Fracture	1
Falls	4/14
Infections	6/14
Thrombosis	0/14
Bladder issues	4/14
Bowel issues	7/14
Body mass index when hospitalized, median (range)	22 (18–34)
Body mass index when discharged, median (range)	26 (19–40)
Weight development in kilograms during hospitalization, median (range)	7 (–11–+22)
Use of pain medication on SRH discharge^a^	8/13
Carriership of multi-resistant bacteria	
Methicillin-resistant *Staphylococcus aureus*	2/14
Extended spectrum beta-lactamase A	8/14
Extended spectrum beta-lactamase and carbapenemase producing bacteria	13/14
Vancomycin-resistant *Enterococcus*	5/14
Infection isolation regimen at hospitalization	
Droplet	7/14
Contact	7/14
Infection isolation regimen on discharge^a^	
Droplet	1/13
Contact	11/13
None	1/13

a One patient was not discharged at the time of data analysis, and therefore not included in the discharge-specific analyses.

### Communication

**Fig. 2:**
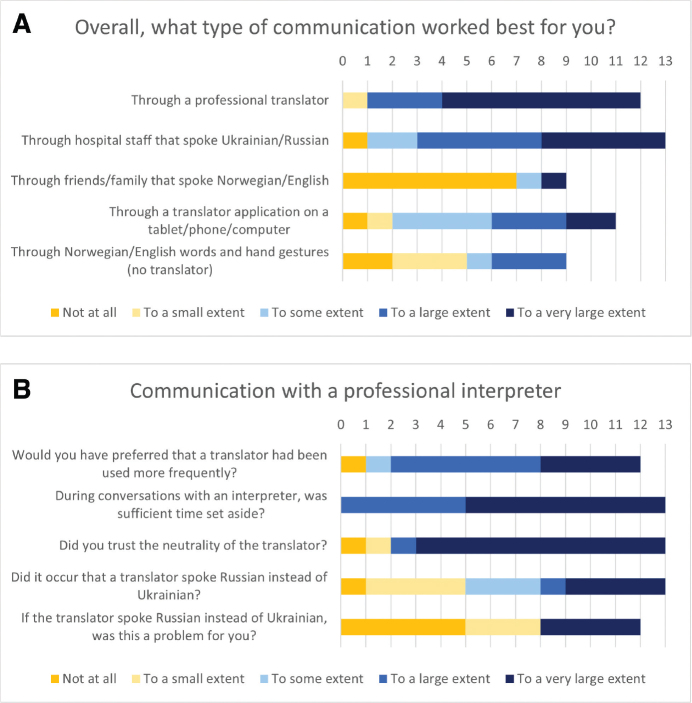
(A) Patients defining what type of communication worked best for them during rehabilitation. (B) Patient experiences of communication with a professional interpreter during rehabilitation.

**Fig. 3:**
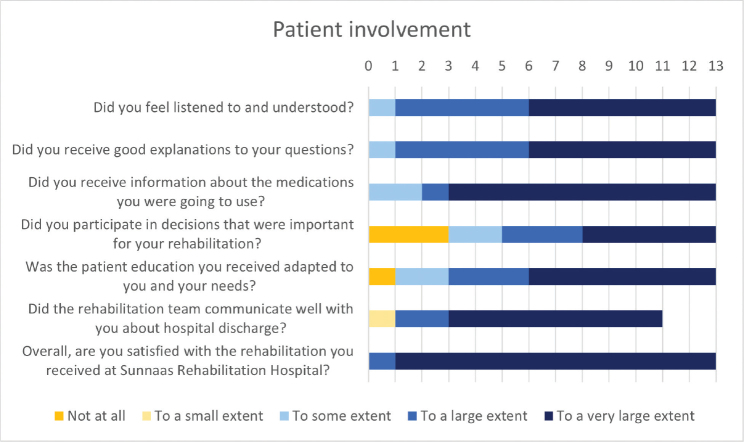
Patient experiences with their own involvement and the rehabilitation process.

All patients were proficient in Ukrainian and Russian. They reported that all professions used PMI, hospital staff, and translation applications for translation (Fig. S1). Communication with doctors was mostly through PMIs. PMIs worked best for patients, closely followed by hospital staff who spoke Ukrainian or Russian (Fig. 2A). Most patients trusted the neutrality of PMIs and wished they had been used more frequently (Fig. 2B). As Ukrainian-speaking PMIs were not easily acquired, nearly all patients experienced Russian-speaking PMIs instead. Four reported this as a great problem, and 1 commented “Do not use Russian interpreters”. Overall, patients felt listened to, understood, and satisfied with the information received (Fig. 3). They reported a high degree of satisfaction with overall communication (Fig. S2).

Health professionals described having to change their communication style towards more clear messages or commands instead of encouraging shared decision-making. They described several practical issues with using PMIs: acquisition, increased time consumption, technical challenges with video/telephone, and requiring specific communication skills. Incorrectly conveyed information led to misunderstandings. They said that PMIs translated better than Google Translate, whose translations were often incorrect and nonsensical – even for simple messages. One health professional said that some patients were better at using Google Translate than others, depending on their dialect and understanding of which phrases were more easily translated. He also stated, “The boy next door [in Ukraine] died…. That day, you should not try to talk through Google Translate, in my opinion. One thing is that they have the right to an interpreter, but one has to be a bit like, and think about ‘what if today is the day they really have something to tell?’…. There is a reason for their right to have it [an interpreter].”

Some health professionals wanted more in-person interpreters, who ideally also worked as in-hospital social educators. One department had several among the nursing staff who spoke Ukrainian or Russian (multilingual staff). This was highlighted as an invaluable resource. They passed on short messages, functioned as conversation partners, had important roles as day-to-day cultural mediators, and repeated the principles for SRH rehabilitation.

### Cultural differences

**Fig. 4:**
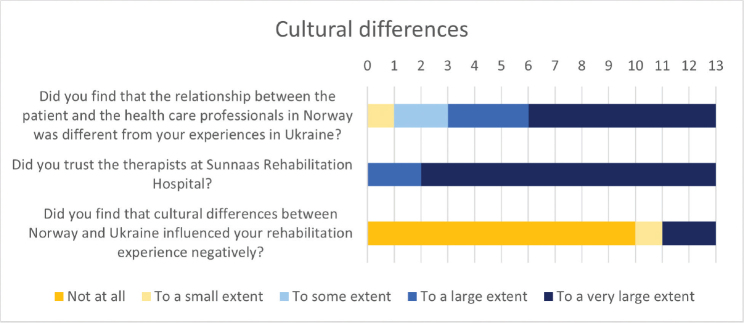
Patient experiences with cultural differences and influence on rehabilitation.

Six patients reported great satisfaction with the food, 5 were neutral and 2 were less content. For other SRH patients in the same department in 2023, 58% reported a large degree of food satisfaction. Although many patients found the relationship between patients and health professionals to be very different from Ukraine, they did not find that this influenced rehabilitation negatively (Fig. 4). They reported high levels of trust in hospital staff and commented that everything was tailored for the patient and that the staff were caring and professional. One participant wrote: “The main difference, in my opinion, was that the doctors gave hardly any prognosis. On the contrary, they asked about my goal, what I wished to achieve, and based on that they created conditions for realization.”

Hospital staff mentioned cultural differences in alcohol consumption, swearing, and acting out. They described patients as having a lot of pride in Ukraine. Some patients became sceptical if the staff smiled a lot, and others lost trust in the Norwegian healthcare system if they underwent an amputation in Norway. Health professionals said that patients expected a more authoritarian healthcare system and that physicians had to confirm instructions from other MDT members. One health professional described difficulties in discussing challenges with urination/defecation. However, the greatest reported cultural difference was the perception of what rehabilitation entails.

### Infection isolation

All patients but 1 were carriers of extended spectrum beta-lactamase- and carbapenemase-producing bacteria. Two were carriers of methicillin-resistant *Staphylococcus aureus* and 4 of vancomycin-resistant *Enterococcus* (see Table II). Upon hospitalization, all patients were placed under infection isolation. One patient was isolated for 6 days. The remaining 13 were isolated throughout their hospital stay.

**Fig. 5:**
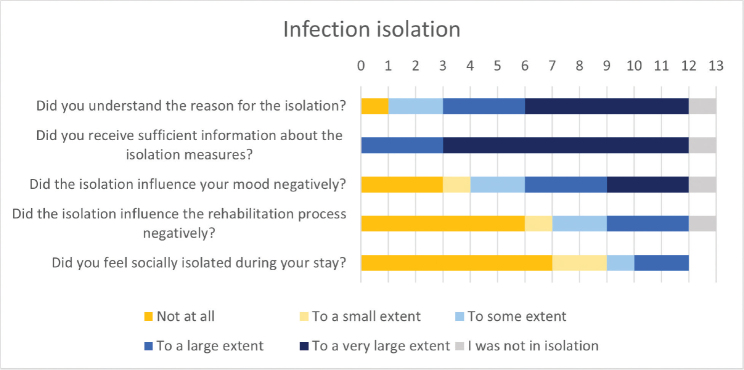
Patient experiences with infection isolation and influence on mood and rehabilitation.

Most patients reported understanding the reason for isolation and receiving sufficient information concerning the isolation measures (Fig. 5). The majority reported that infection isolation negatively influenced their mood, but not the rehabilitation process. Most did not report feeling socially isolated.

Health professionals were under the impression that patients did not understand the cause of infection isolation or the potential harm of carrying MDROs. They described several challenges with infection isolation. For example, patients could not use several hospital facilities, which may have made inpatient rehabilitation seem less meaningful. Lower activity levels could also reduce appetite. The combination of handling exercise equipment, using PMIs, and infection isolation equipment entailed more coordination and time consumption. One health professional said, “[You] spend more time getting less done, it feels like”.

Several health professionals were especially concerned about patient mental health, describing the combination of psychological trauma, language barriers, and isolation – where those entering had covered faces – as particularly detrimental. One health professional noted that the hospital guidelines did not weigh the benefits well enough against the disadvantages in terms of the psychological strain that isolation caused patients. Some also questioned whether SRH had stricter guidelines than other hospitals.

With time, some professionals were allowed to have conversations in patient rooms without face masks, which they said helped. Altered infection isolation routines also gave some patients fewer restrictions. Health professionals said they took on more of the burden by accompanying patients so that they could have more freedom within the hospital to exercise at the gym and stay in common areas. Relatives staying with patients in their rooms also reportedly counteracted social isolation. Over time, hospital staff found they became less uncertain regarding infection prevention guidelines and more skilled at facilitating and coordinating rehabilitation for patients in isolation. They had better exercise equipment in the patient isolation areas, and performed more rehabilitation outdoors (i.e., training, activities, and excursions).

### Psychosocial load

All patients had a first-time consultation with a psychologist, and most did not want further psychological treatment. Seven had more than 1 conversation with a psychologist; however, 1 was mandatory after an incident involving physical violence towards another Ukrainian patient. When asked about their mood during hospitalization, one patient reported “average”, while 5 reported “good” and 7 “excellent”. Concerns regarding life in Norway were sparse and not reported to influence the rehabilitation process negatively (Fig. S3). Four had concerns related to the time after discharge, with free-text comments noting “rehabilitation” and “No clear deadlines. You need to wait for a long time.” The ongoing war had a considerable negative influence on the moods of several patients. One commented feeling depressed because their family was in Ukraine. Patients were split when asked whether the war negatively influenced the rehabilitation process, but most did not believe so. They expressed a desire to work hard in order to contribute to defending Ukraine. They appreciated the actions of staff to improve quality of life. One wrote: “There were several trips to museums and to Oslo itself, I liked that a lot and am grateful to the arrangers. There were also concerts and different activities in the evenings, especially around holidays, and a big Christmas stocking filled with candy.”

Health professionals said that some patients were affected by many years of war, since 2014. Several had trouble sleeping. They emphasized that the patients live with ongoing trauma that overshadows everything else. One health professional said that many confirmed having a hard time, with constant messages about dead friends, but “they could not go into what had been, because then life does not add up, in a way”. Some said that patients could be crudely classified into 2 groups: those with military education (whom they assumed had been more prepared for war and trauma) and those who were civilians or fought without military education. The latter could have problems following timetables and rules. Some reported that patients had an intense hatred towards Russia, not wanting to interact with Russian staff or patients, while Russian-speaking staff from other countries were more easily accepted. One health professional who was half Russian found some patients intimidating, while others seemed afraid of her. Nevertheless, she felt that she achieved a good treatment alliance with them over time.

Health professionals found that multilingual staff seemed to make patients feel safer and that they functioned as conversation partners. Spending time with other Ukrainians also seemed beneficial for patients’ mental health. They also noted that patients responded well to practical tasks that gave a mental break from the war, such as cooking, excursions, and sessions with physical therapists or occupational therapists. Getting their family to Norway and knowing they were safe made it possible for some to focus on their own rehabilitation.

### The rehabilitation process

The median number of multidisciplinary meetings was 3 (range: 1–7), implying 1 meeting every 5 weeks. Eleven patients had defined overall goals in their treatment plans, which all included some version of being able to walk. While 11 required assistance in personal activities of daily living when they arrived, only 1 did on discharge. Four required assistance from a primary healthcare nursing facility in wound care, 1 required assistance in bowel functioning, and 3 required assistance in the administration of medications. All patients required physical therapy (Table III). Six had more medical treatment planned at a hospital (e.g., orthopaedic surgery or facial reconstruction).

**Table III:** Further needs after discharge from Sunnaas Rehabilitation Hospital^a^

Primary health care physical therapist	13/13
Walking aids	12/13
Aiding tools at home	8/13
Orthopaedic engineer	8/13
Planned medical treatment at a hospital (specialized healthcare)	6/13
Primary health care occupational therapist	4/13
Primary health care nursing facility	4/13
Help in personal activities of daily living	1/13
Primary health care psychiatric nurse	1/13
Primary health care rehabilitation team	1/13

a One patient was not discharged at the time of data analysis, and therefore not included in these analyses.

Eight patients reported having participated in decisions important to their rehabilitation to a large extent, and 10 that patient education was adapted to their needs (see Fig. 3). Other SRH patients reported 87% and 83%, respectively. All but 1 patient found that the rehabilitation team communicated well regarding hospital discharge, and 11/13 reported receiving information concerning their medication. In comparison, other SRH patients reported 92% and 46%, respectively. In the CSQ-8, the median total satisfaction score was 30/32 (range: 24–32; Fig. 6). In the custom-made questionnaire, 12 patients were very satisfied and 1 was satisfied with the overall rehabilitation they received, compared with 60% and 35% for other SRH patients, respectively.

**Fig. 6:**
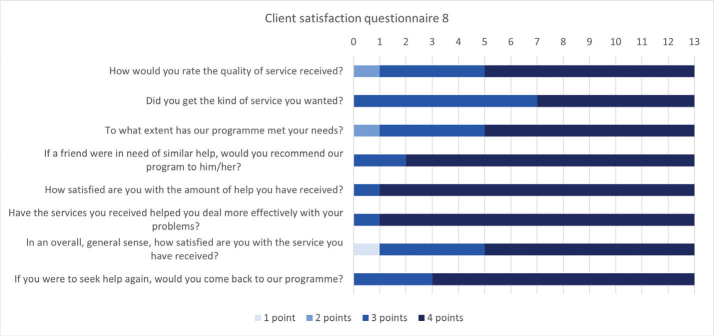
Client satisfaction questionnaire 8. One point indicates the lowest level of satisfaction, and 4 points indicate the highest level of satisfaction.

Hospital staff reported that patients often did not understand what was expected of them in the rehabilitation process. Patients expected authoritarian health professionals and passive rehabilitation measures such as massage. At first, they were confused by questions such as “What is important to you?”. Patients also required repeated explanations concerning the rehabilitation process from multilingual health professionals. One health professional said that long-term institutionalization could contribute to passivity. Others noted that one could not expect someone who is emotionally and mentally at war to be able to take ownership of their rehabilitation process.

Health professionals were unanimous in stating that the complex, multidisciplinary rehabilitation provided was useful and something that these patients needed. They said the rehabilitation quality improved with experience gained and felt they had gone to great lengths for their patients. When solving practical issues, they described a “system lightness” and flexibility in the hospital administration that provided an increased feeling of mastery and meaning.

Health professionals described transitions as particularly vulnerable. Immigrant regulations and unclear guidelines frequently made the discharge location uncertain until the last moment, which made it difficult to involve local services. Although some said that ARCs did a good job, others described them as unorganized, with unpredictability in what they could offer, challenging communication and mostly serving as “storage”. Staff could not know whether recommendations from SRH were forwarded to the primary healthcare providers when patients received a residence. Some mentioned that patients felt safer if they were discharged to the same ARC. Nine patients had experienced follow-up in the primary healthcare services of different municipalities when filling out the questionnaire. Most were satisfied and reported sufficient follow-up after discharge, but 3 did not (Fig. S4).

### Impact on health professionals

Health professionals said that this war felt closer to them because of the tragedy they discovered while treating these patients. Although it helped to know that they contributed, they required more debriefs and support from colleagues. They lacked prior experience with war medicine and described expectations to perform above their current level of competence with such complex war trauma. They wanted more cooperation with other hospitals that treated MEDEVAC patients.

## DISCUSSION

This combined methods study has assessed the rehabilitation provided to Ukrainian citizens evacuated through the MEDEVAC programme. The focus was on how communication, cultural differences, infection isolation, psychosocial load, and attitudes toward the rehabilitation process influenced the rehabilitation experience. Patients and MDTs showed flexibility in the rehabilitation process, adapted well to many foreign concepts, and described the rehabilitation as useful. Their experiences provide valuable insights into how patients with other cultural backgrounds should be treated during rehabilitation.

### Communication

Communication is one of the pillars of rehabilitation, and others have demonstrated that rehabilitation with language interpretation is useful (30). In the present study, patients expressed the highest degree of satisfaction with PMIs. As in another study (4), health professionals reported practical issues with using PMIs but emphasized the need for them. For the Ukrainian MEDEVAC patients, difficult war experiences can be associated with the Russian language. Thus, as reported here, Russian PMIs may add to the psychological burden for patients. Despite this, trust in PMI neutrality was reportedly high.

Both groups were less satisfied with translation applications, whose utility may depend on the individual. Although such applications may aid day-to-day messaging, one must consider that others have reported low accuracy and some serious translation errors (31).

While others reported higher patient satisfaction with PMIs when compared with multilingual hospital staff (32), satisfaction with the latter was almost as high as for PMIs in this study. However, these employees contributed with more than just interpreting, which possibly influenced the high levels of satisfaction. They served as conversation partners and cultural mediators while also repeatedly explaining the rehabilitation process and solving practical issues.

Non-verbal communication, such as facial expressions and demonstrations, serves an important role during therapy, especially when language barriers exist. Consequentially, as mentioned by health professionals, infection prevention with face masks may have negatively influenced communication.

### Cultural differences

Cultural differences might affect the dynamics of trust, respect, and collaboration between patients and health professionals. For instance, differing cultural norms regarding authority, emotional expression, or decision-making might influence how patients and staff interact. Health professionals said that patients expected an authoritative manner of communication and that some lost trust in the Norwegian healthcare system if their amputation occurred in Norway. Patients reported high levels of trust in the health professionals despite great cultural differences. They were positive about patient involvement and caring health professionals. This may have been influenced by multilingual health professionals also acting as cultural mediators. By addressing practical, emotional, and cultural needs that enhanced the therapeutic process, they may have bridged gaps in understanding, contributed to stronger therapeutic relationships, and facilitated more personalized care. One could argue that staff speaking the same language as patients may experience difficulties staying objective and professional. Others have discouraged *ad hoc* interpreters due to a lack of training and patients fearing for their confidentiality in a migrant community (3). However, this was not a reported issue in this study. Others have described cultural brokers as useful in helping health professionals adjust to the patient’s culture (4). Staff might also have benefited more from their cultural mediation, if this competency had been further integrated into MDTs.

### Infection isolation

Although patients in infection isolation have the right to equal treatment, practical issues in rehabilitation occur. Examples that may influence the rehabilitation experience include limited access to shared facilities, group therapy sessions, and social interactions. Patient understanding of infection isolation may have been influenced by both communication and cultural differences. In this study, health professionals were particularly concerned about the consequences for patient mental health, and patients reported a negative impact on their mood. One previous study reported that infection isolation did not influence psychological outcomes during rehabilitation (33). However, the study population was also small (*n* = 16) and was not involved in a war (33). Over time, health professionals found that they improved at providing rehabilitation despite infection isolation, for instance by bringing more equipment into the patient room or exercising outdoors. They became more skilled at upholding infection prevention regimens for patients using common facilities.

### Psychosocial load

There are many sources of high psychosocial load in this patient group. Many have war trauma. The ongoing stress of war is exemplified by a patient displaying fear simply because a nurse speaks Russian. Health professionals said continuous war updates and worries about loved ones seemed to overshadow everything else for patients. There were also practical issues in Norway, such as their refugee status, personal economy, and housing. Their lack of any social network in Norway, as well as infection prevention, may cause social isolation. Furthermore, mental health may be negatively influenced by long-term illness and pain. Health professionals said that many patients had sleep issues and seemed affected by many years of war. Nevertheless, most patients reported their mood as being good to excellent during hospitalization, which may imply that health personnel overestimated the psychological effects of their situation. The potential sources of error should be acknowledged, such as recall bias, selection bias (where those with the poorest mental health may not have participated), and underreporting symptoms (because mental health is more taboo in Ukraine) (16). Patients may have wished to prioritize physical recovery and underreported mental health symptoms because they considered them secondary. However, the efforts of health professionals to improve quality of life during admission may have also made an impact.

### The rehabilitation process

Patients may have differing perspectives concerning the level of involvement required in their own care, or focused on long-term recovery vs immediate relief. Despite health personnel reporting that patients had trouble understanding the concept of multidisciplinarity and ICF-based rehabilitation, patients reported high satisfaction. Ukraine is currently working on implementing this type of rehabilitation by introducing the ICF model and training rehabilitation professions (19). The Olena Zelenska initiative “Barrier-free” aims to give all Ukrainians equal opportunities, also addressing the terminology for people with disabilities (34). Despite being initiated recently, the campaign might have already made an impact. Patients showed flexibility in adapting and provided positive feedback for rehabilitation. Participating in an environment where these principles are well established and seeing others achieve results may also increase faith in the method. The importance of cultural mediators, who repeatedly explained the process, was highlighted by several health professionals. Gaps may also have been bridged as health professionals adapted to better suit the patient group and adjusted their methods for communication. The present results indicate that Ukrainians can be open to a less authoritative healthcare system with more patient involvement, which would represent an important ongoing paradigm shift in Ukraine.

Health professionals stated that they could not expect the same degree of owning one’s rehabilitation process from these patients as they were mentally “at home”. Rehabilitation focuses on life after discharge and can be challenging with evacuated patients because uncertainty makes it difficult to plan a further course. Should their long-term goals be related to life in Ukraine or integration into Norway? In which country should the team plan follow-up? Further adding to this uncertainty were ambiguities and frequent changes in Norwegian regulations for the rights of MEDEVAC patients.

### Methodological considerations

Limitations in this study include potential recall bias for patients and health professionals, possible reporter bias in medical files, small sample size, 1 department in 1 institution being studied, and the vulnerability to human error as only 1 researcher (MRK) performed data plotting and transcription. Only descriptive statistics were feasible, and further studies are needed to build on these results. Both participant groups have a risk of selection bias, as satisfied patients are more likely to participate. Although knowing the researchers and depending on hospital services may provide a sense of obligation to participate and report falsely high satisfaction, the consent form clearly stated voluntary participation with no influence on treatment or employment. While some had been admitted for only a month when they replied to the questionnaire, this should be sufficient time to obtain an impression of the rehabilitation provided. Moreover, qualitative analyses are vulnerable to subjectivity, representativeness, and verifiability (28). As a physician at SRH, MRK participated in the rehabilitation of Ukrainian MEDEVAC patients, facilitating the use of clinical experiences in data collection and analysis. Although important in semi-structured interviews, this also has a risk of leading questions and inferring too quickly from participant statements (27). There are also potential sources of error in filling out the CSQ-8 because the alternatives indicating the highest and lowest degrees of satisfaction switch places for each question.

### Conclusion

In conclusion, evacuating patients from a country at war and providing specialized rehabilitation was feasible and valuable for both patients and health professionals, despite the challenges posed by the 5 fields assessed in this paper (i.e., communication challenges, cultural differences, infection isolation, increased psychosocial load with uncertainties from ongoing war, and different expectations for the rehabilitation process). Across all fields, valuable clinical experience in war trauma rehabilitation was gained. This is an important part of civil protection, and relevant to a vast number of patients. Patients and health professionals displayed flexibility and willingness to adapt. Patients reported high levels of satisfaction with the goal-oriented, ICF-based rehabilitation provided. In this process, the frequent use of professional interpreters was important. Staff members with shared language and cultural backgrounds also served crucial roles not only as translators but also as cultural mediators, and helped bridge gaps to improve the rehabilitation experience.

## Supplementary Material


