# Supplementary material for: UKRAINIAN WAR TRAUMA PATIENTS ABROAD: THE REHABILITATION PROCESS IN LIGHT OF LANGUAGE BARRIERS, CULTURAL DIFFERENCES, WAR, AND INFECTION ISOLATION
Source: J Rehabil Med. 2025 Aug 11;57:42929. doi: 10.2340/jrm.v57.42929 (PMC12359811; doi:10.2340/jrm.v57.42929)

Supplementary material has been published as submitted. It has not been copyedited, or typeset by Journal of Rehabilitation Medicine

Fig. S1. Patient experiences with communication means used by different health professions and in multidisciplinary meetings. i) nurses. ii) doctors. iii) therapists. iv) multidisciplinary meetings.

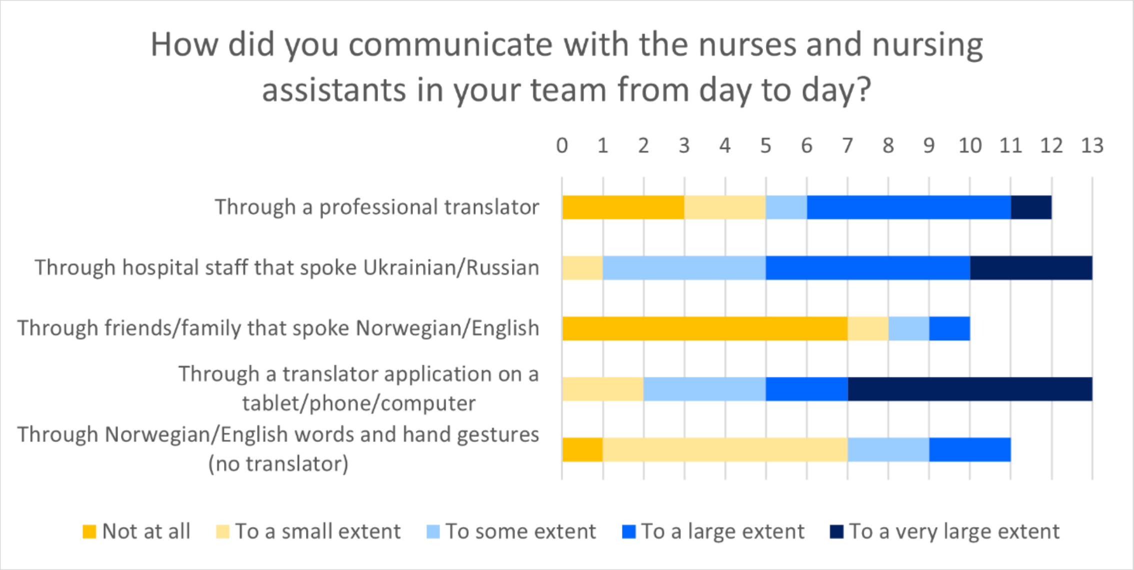

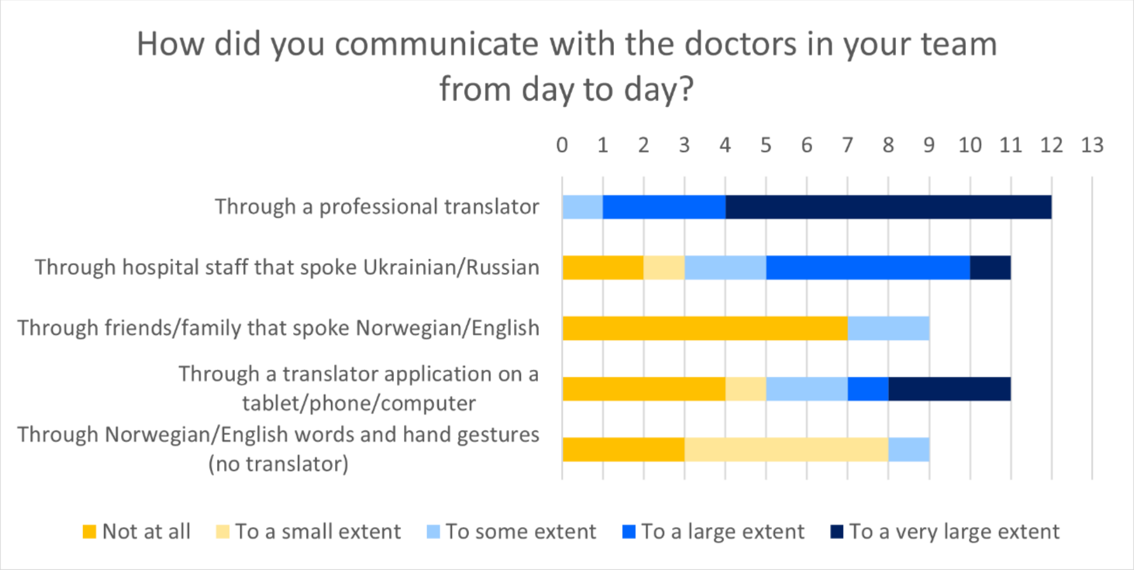

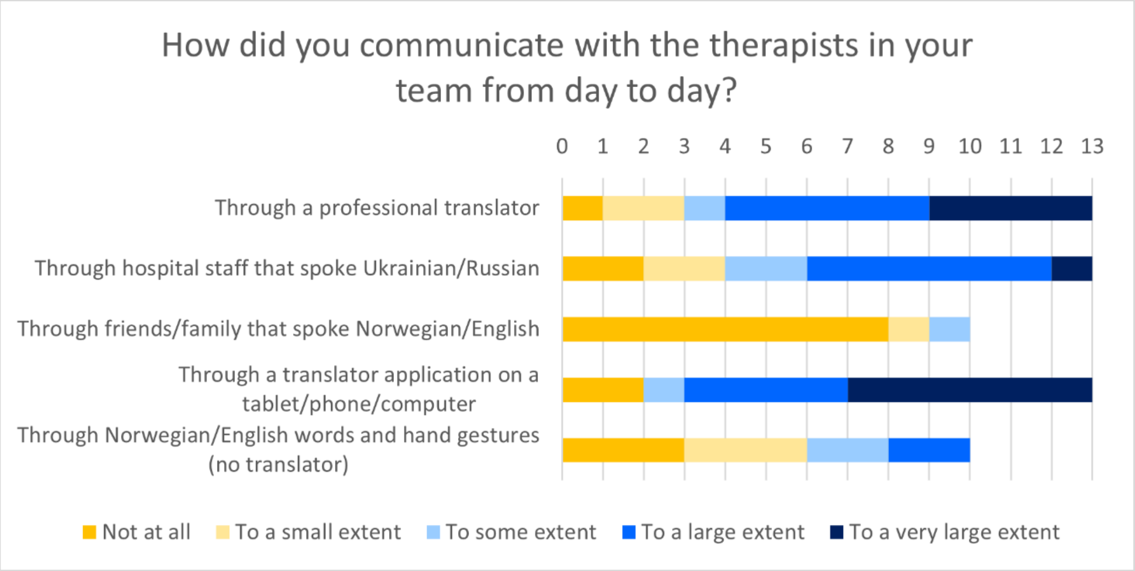

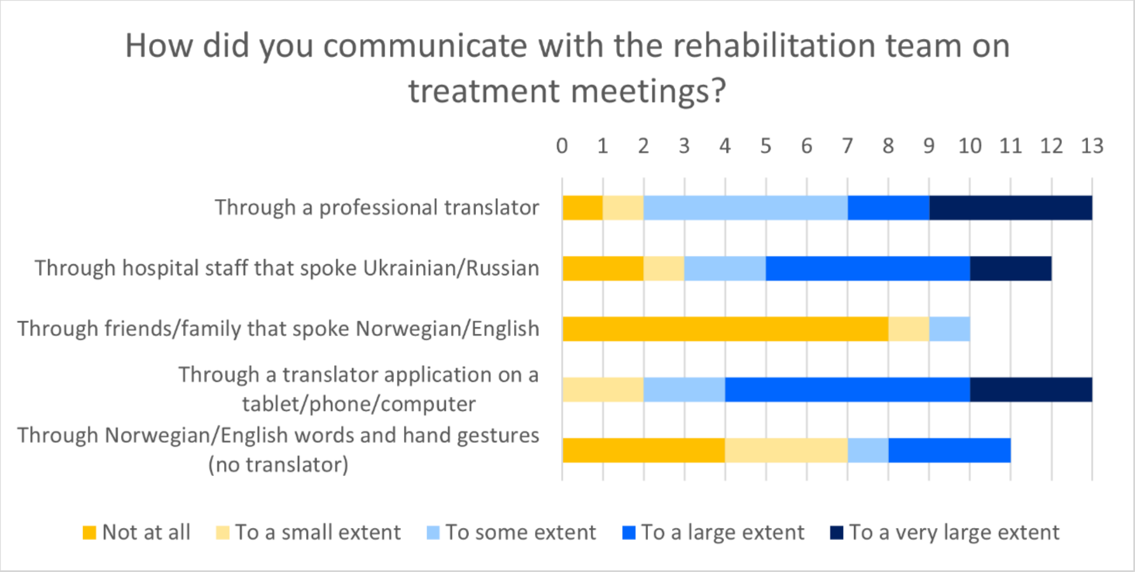

Fig. S2. Patient satisfaction with communication during rehabilitation.

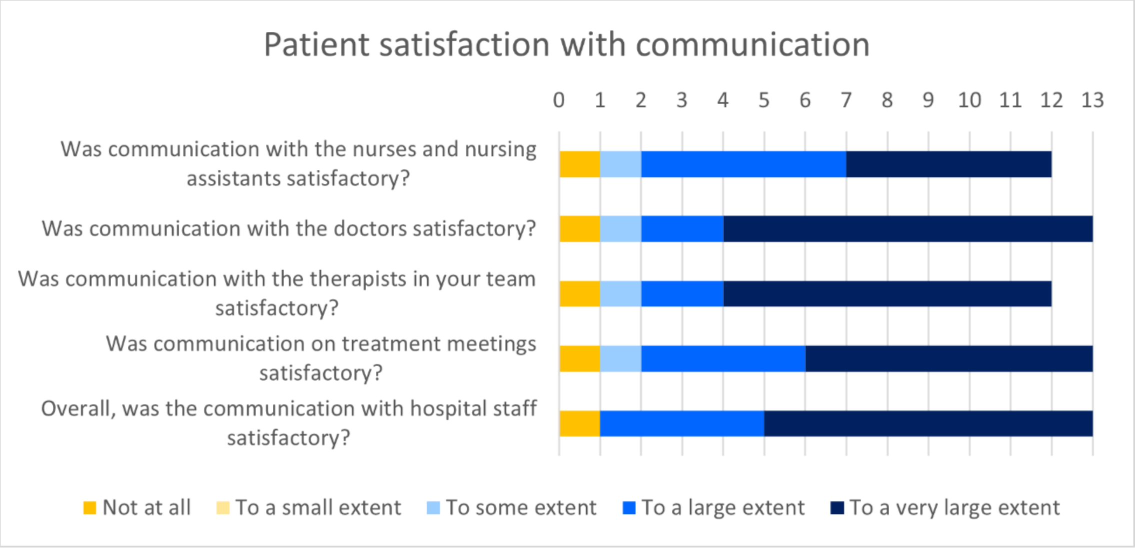

Fig. S3. i) Patients' social concerns about life in Norway, and their influence on rehabilitation.

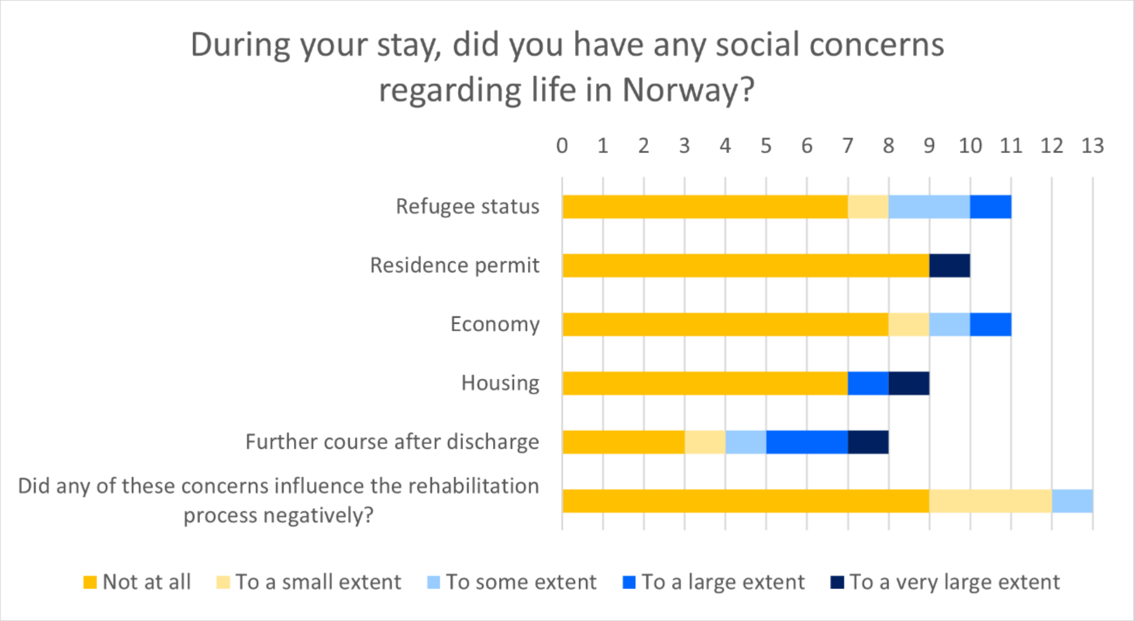

Fig. S3. ii) How patients and rehabilitation were influenced by the war in Ukraine.

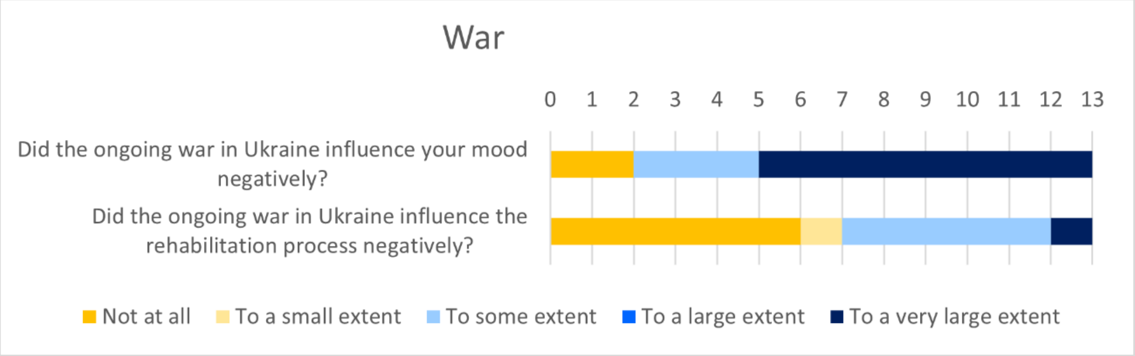

Fig. S4. Patient experiences with follow-up after discharge.

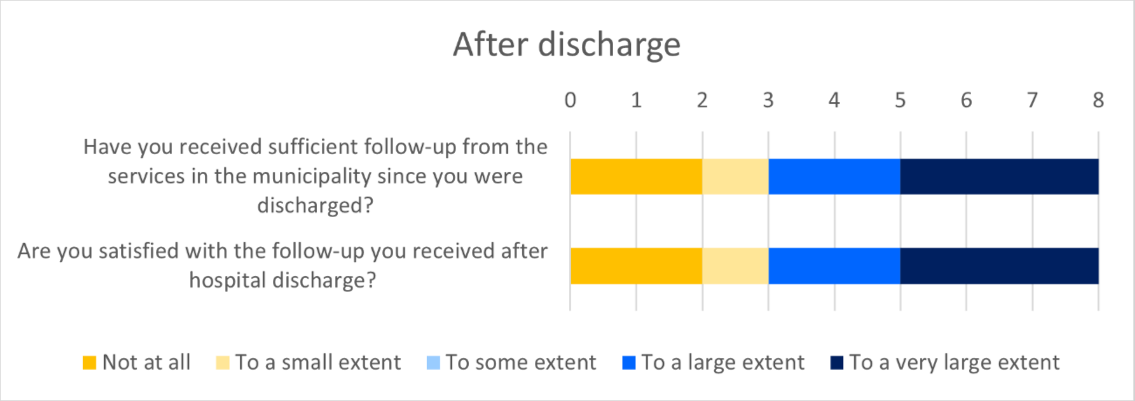

Supplement: Supplementary file 1 [file JRM-57-42929-s1.pdf]
